# Post-traumatic stress disorder symptoms in high-risk pregnant women during the perinatal period: a longitudinal study

**DOI:** 10.3389/fpsyg.2026.1795171

**Published:** 2026-08-13

**Authors:** Yanhong Wang, Sufei Wei, Ruirui Wang, Yaxin Chen, Qi Jin, Ke Zhang, Xiaoxia Liu

**Affiliations:** 1 School of Nursing, Lanzhou University, Lanzhou, Gansu, China; 2 Tianshui First People's Hospital, Tianshui, Gansu, China; 3 Gansu Provincial Hospital of Traditional Chinese Medicine, Lanzhou, Gansu, China

**Keywords:** group-based trajectory modeling, high-risk pregnancies, longitudinal design, perinatal mental health, post-traumatic stress disorder

## Abstract

**Objective:**

This study employed a longitudinal design to investigate perinatal post-traumatic stress disorder in high-risk pregnant women across multiple time points, aiming to identify optimal timing for intervention.

**Methods:**

A longitudinal research design was used to assess the P-PTSD inventory scores at 1 month after diagnosis of high-risk pregnancy (T_0_), 3 months after diagnosis (T_1_), 6 months after diagnosis (T_2_), and 1 month postpartum (T_3_), to analyse the overall trend of P-PTSD using generalized estimating equations, and to determine the heterogeneity of P-PTSD in different characteristics of high-risk pregnancies by using a group trajectory model.

**Results:**

Among 245 high-risk pregnant women, the positive detection rates of P-PTSD at T0–T3 were 12.7, 29.8, 31.4, and 35.9%. Generalized estimating equations revealed a significant overall upward trend (χ^2^ = 64.02, *p* < 0.001). Group-based trajectory modeling identified three distinct trajectory classes—low-risk stable (36.1%), moderate-risk worsening (46.8%), and high-risk fluctuating (17.1%)—with the 3-group solution (polynomial order 1, 2, 2) selected based on the lowest BIC (−3438.08) and acceptable classification accuracy (Entropy = 0.71). Multinomial logistic regression identified several independent predictors of trajectory membership, including monthly household income (e.g., 6,001–9,000 yuan vs. lower income: OR = 0.19, 95% CI: 0.04–0.86) and prior trauma history (no vs. yes: OR = 0.17, 95% CI: 0.03–0.93).

**Conclusion:**

This study found through longitudinal tracking that P-PTSD symptoms in high-risk pregnant women generally worsened over time, and that there was significant heterogeneity in the development trajectory of symptoms in this group. Socioeconomic status, perinatal-related factors, and trauma experiences are the core predictive variables distinguishing different symptom development trajectories.

## 1 Introduction

The proportion of high-risk pregnancies has risen as a result of the delay in the maternal age at childbearing, the development of assisted reproductive technology and the adjustment of China’s reproductive policy (three-child policy) (Grekin and O'Hara, 2014). High-risk Pregnancy (HRP) is a pregnancy with unfavorable factors or comorbidities that may endanger the pregnant woman, the fetus, or the newborn, or result in obstructed labor, and is a pregnancy that requires close and supervised care (Giarratano et al., 2015). According to the World Health Organization, the incidence of high-risk pregnancies is about 6 to 33% globally (Mäkelä et al., 2023). High-risk pregnant women are often subject to higher levels of obstetric intervention and more complex care, including lifestyle, medication, and even hospitalization. They experience more uncertainty, greater psychological stress, and a reduced sense of well-being, making them face an elevated risk of perinatal post-traumatic stress disorder (P-PTSD) symptoms (Levey et al., 2018). PTSD refers to a category of delayed psychological disorders that occur after the organism has been exposed to or confronted with a major stressful event (Johansen et al., 2022; Sleet, 2018). PTSD is dominated by the three core symptoms of re-experiencing, avoidance/numbness, and hypervigilance, and symptoms persist for more than 1 month (Bergunde et al., 2023). P-PTSD refers to a diagnosis of PTSD from conception to 6 months postpartum. Studies have shown that the percentage of high-risk pregnant women with P-PTSD ranges from 18.5 to 43.0% (Webb and Ayers, 2015). Inadequate diagnosis and treatment are associated with an increased risk of adverse maternal and infant outcomes such as postpartum depression, substance abuse, and preterm birth (Beck and Watson, 2010).

The severity and core symptom profile of P-PTSD in high-risk pregnant women show dynamic changes at different stages of the perinatal period. As the perinatal period progresses, the nature and intensity of the stressor may change, affecting the severity and presentation of P-PTSD symptoms (Onoye et al., 2013; Yildiz et al., 2017). For example, in early pregnancy, high-risk pregnant women may be primarily concerned about the development of the fetus and their health, while in late pregnancy and after delivery, fear of the birthing process, labor pains, and concerns about the health of the newborn become the main sources of stress. Vignato et al., found that 30.4% (84/276) of pregnant women experienced P-PTSD symptoms at different stages of the perinatal period (early, middle, and late pregnancy and 6 weeks postpartum), with prevalence rates of 32.2% (19/59), 32.3% (30/93), 28.6% (22/77), and 27.7% (13/47), respectively (Vignato et al., 2018). A multicenter study by Onoye et al. examined the linear changes in P-PTSD from 3 months of gestation to postpartum, and the trend in scores showed an increase and then a decrease, with a significant “peak” in late gestation and a gradual decrease in the postpartum period (Orovou et al., 2022). However, these studies did not distinguish between normal and high-risk pregnancies, which may explain large differences in outcomes (Paulson et al., 2023; Muzik et al., 2016; Dikmen-Yildiz et al., 2018; Malaju et al., 2022; Wang et al., 2023). In the case of high-risk pregnancies, there are large differences in attitudes between healthcare professionals and pregnant women regarding the perceived risk of stressors and risk factors in pregnancy, resulting in a self-perceived “anchoring effect” that pulls risk estimates in the direction of one’s preconceptions. This results in the “high risk” label may function as an additional psychological burden, potentially contributing to P-PTSD symptoms. Therefore, categorizing high-risk pregnant women into different categories can objectively provide an overall assessment of the predictors of P-PTSD and accurately reflect the level of P-PTSD in high-risk pregnant women. Therefore, a longitudinal study design is needed to explore the mechanisms of psychological change throughout the perinatal period in high-risk pregnant women at risk for P-PTSD.

Research has found that the educational level of pregnant women, stress/trauma exposure during pregnancy, delivery anxiety, social support, and comorbid depression all have an impact on the symptom trajectory of P-PTSD. For example, educational attainment may influence the way individuals cognitively assess stressful events (e.g., childbirth) and the choice of coping strategies. Higher cognitive ability may contribute to greater flexibility in responding to challenges, adopting more positive coping styles, and reducing the risk of perceiving the birth experience as traumatic (Vignato et al., 2018). Most of the existing studies on factors influencing P-PTSD symptom trajectories have not explored multifactorial interactions based on a systems theoretical framework. Exploring the role of only a single factor, such as social support or psychological resilience, lacks a holistic grasp of multifactorial interactions, resulting in findings with limited explanatory power. Recent studies on the psychological adjustment during the perinatal period under conditions of large-scale stress have shown that, whether it is a sudden stress such as a global pandemic, or a persistent pregnancy-related threat such as high-risk pregnancy, the impact of situational stressors on different women is not uniform; its effects are mediated through individual psychological resources and social support systems (La Rosa et al., 2025; La Rosa et al., 2024). This perspective is consistent with the SMS model and provides a useful reference for understanding the potential heterogeneity of the P-PTSD trajectory. Therefore, guided by the Stress Systematics Model (SMS) as the theoretical framework, this study conducted a longitudinal investigation of perinatal post-traumatic stress disorder and its three core symptoms in high-risk pregnant women across different time points, aiming to identify the optimal timing for intervention and to provide evidence for the development of targeted and effective intervention strategies (Forray et al., 2009).

## 2 Methods

### 2.1 The theoretical framework of the study

**Figure 1:**
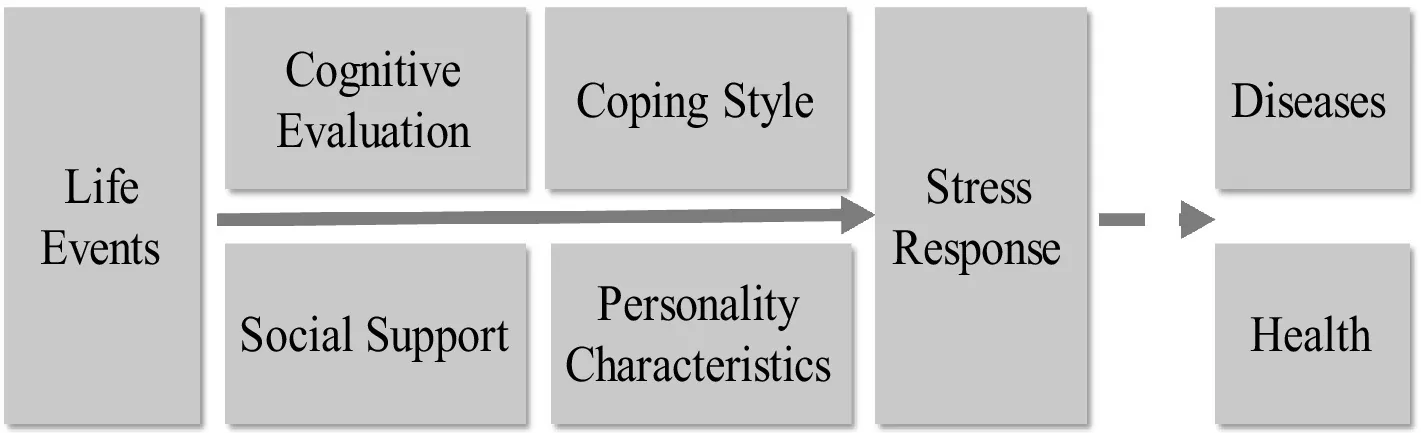
The SMS schema.

The SMS was developed by the Chinese scholar Jiang (2014), it is based on a comprehensive cognitive stress theory. This theory posits that stress is a multifactorial process. It asserts that when an individual’s own needs and the ability to satisfy those needs are in conflict, the organism can maintain a relative equilibrium by regulating physiological and psychological responses. This enables the organism to adapt to the stressors that arise, see Figure 1.

**Figure 2:**
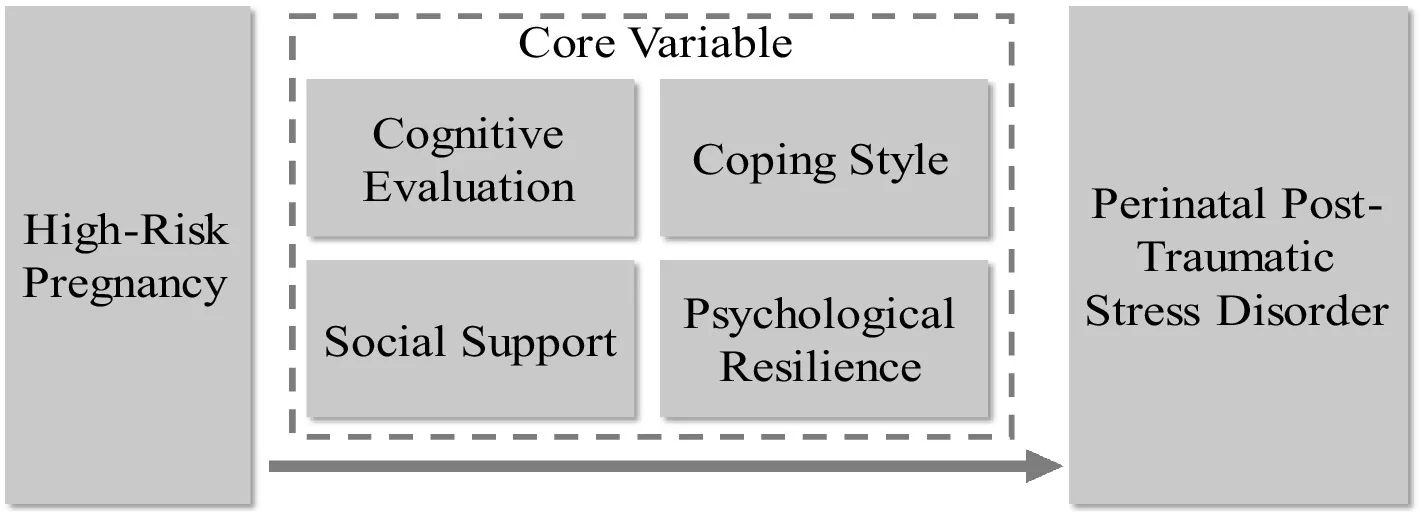
The P-PTSD psychological stress process.

Cognitive evaluation refers to an individual’s self-estimation of the nature and severity of a stressor and the harm it may cause to them. Coping style is an individual’s behavioral response to a stressor, and to their own instability arising from that stressor. Social Support refers to the extent to which individuals are mentally or physically connected to social networks, including individuals such as family and friends, and organisations such as families and workplaces (Jiang, 2013). Treglown et al. (2016) proposed psychological resilience as a personality characteristic. Positive psychology suggests that an individual’s psychological resilience depends on a combination of emotional perception, emotional activation, and personality traits, and that it is a positive adaptive process that enables active self-adaptation. Numerous studies have confirmed that psychological resilience has a significant impact on the development of PTSD (Bai and Zhao, 2022; Ma, 2023; Zhao et al., 2023). In addition, another study verified that the degree of psychological resilience was significantly associated with P-PTSD over time (*p* < 0.05) (Wang et al., 2022). Therefore, the present study adopted psychological resilience as a measure of personality characteristics in at-risk pregnant women, and proposed a research hypothesis that at-risk pregnant women with different levels of psychological resilience may develop different longitudinal developmental trajectories. And a prediction model for the developmental trajectory of P-PTSD in high-risk pregnant women was constructed (see Figure 2).

### 2.2 Operational definitions

#### 2.2.1 The high-risk pregnant women

According to the Norms for Pregnancy Risk Assessment and Management and the Implementation Programme of the Action Plan for Strengthening Maternal and Infant Safety in Gansu Province (2021–2025) (Gansu Health Commission, 2021), the pregnant women be classified into five colors levels—green, yellow, orange, red, and purple—according to their basic conditions and pregnancy complications. “Green” indicates normal pregnancy; “Yellow” indicates general risk and that pregnancy complications are mild and stable; “Orange” indicates medium risk and that pregnancy complications pose a threat to mother and infant safety; “Red” indicates high risk and that pregnant women suffer from serious pregnancy complications, continued pregnancy may endanger the life of mother and infant; and “Purple” indicates that the pregnant women have infectious diseases such as viral hepatitis, syphilis, HIV/AIDS, tuberculosis, serious infectious pneumonia, or a specific viral infection (“Purple” markings for pregnant women may be accompanied by other colours). Therefore, in this study, pregnant women who were classified as “yellow level or above” after examination by an obstetrician-gynaecologist were categorized as high-risk pregnant women.

#### 2.2.2 The P-PTSD symptoms

Chinese scholars generally consider the perinatal period to be from 28 weeks of gestation to 1 week postpartum; in developed countries, the definition of the perinatal period has been expanded to include pregnancy to 1 year postpartum. The American College of Obstetricians and Gynecologists (ACOG) defined the scope of P-PTSD as the physical and psychological stress reactions from the beginning of pregnancy to 12 months postpartum (Pinto et al., 2023). According to a conceptual analysis (Webb and Ayers, 2015), P-PTSD were defined as a diagnosis of PTSD from conception to 6 months postpartum with symptoms persisting for more than 1 month, with antecedents that included traumatic events. The diagnosis of a high-risk pregnancy is itself a significant source of potential traumatic stress. The first month after childbirth is a critical period for mothers facing stress related to the health of their newborns, their own physical recovery and adapting to their new role. Given the follow-up design of this study and the time point for diagnosing high-risk pregnancies, this study defines perinatal procedures as the period from the diagnosis of high-risk pregnancy to 1 month postpartum. On this basis, P-PTSD was defined in this study as the occurrence of maternal symptoms consistent with the core symptom clusters of PTSD (re-experiencing, avoidance/numbing, high alertness) from the diagnosis of high-risk pregnancy to 1 month postpartum.

### 2.3 Study design and participants

*Sample size calculation*. The required sample size was estimated using the formula for cross-sectional surveys based on population proportion estimation: N = $\frac{Z_{1-\alpha /2}^{2}\pi (1-\pi )}{\delta^{2}}$ . Based on a previous study reporting a P-PTSD prevalence of 15.4% among high-risk pregnant women, with a permissible error of 5% and a two-sided *α* of 0.05, the initial estimated sample size was 201. Accounting for a 15% attrition rate, the target sample size was set at 236. The present study ultimately enrolled 245 participants, exceeding this requirement.

*Participant recruitment*. A longitudinal study design was employed to prospectively track the incidence of P-PTSD and its influencing factors among high-risk pregnant women receiving perinatal care at a tertiary hospital (classified as 3A in China) in Lanzhou. The inclusion criteria were as follows: (1) Age ≥18 years; (2) Pregnancy risk assessment: “Yellow,” “Orange,” “Red,” “Purple”; (3) Gestational age ≤14^+6^weeks; (4) Intact cognitive and behavioral abilities with fluency in spoken and written Chinese. Women who combined with malignancies, severe mental disorders or other major systemic diseases (chronic renal failure, chronic heart failure, etc.) were excluded from the study.

#### 2.3.1 Research tools

*PTSD Checklist-Civilian Version (PCL-C)*. The 17-item civilian universal version aligns with PTSD diagnostic criteria from the Diagnostic and Statistical Manual of Mental Disorders, Fourth Edition (DSM-IV) (Ruggiero et al., 2003). In pregnant populations, this scale demonstrates a sensitivity of 86% and specificity of 63% (Gelaye et al., 2017). Items are rated on a 5-point Likert scale, with total scores ranging from 17 to 85. Higher scores indicate greater symptom severity, with individual symptoms considered clinically significant when item scores exceed 3 points. The scale evaluates three symptom clusters: Re-experiencing; Avoidance/numbing; Hyperarousal. The validated Chinese version exhibits good psychometric properties, with Cronbach’s *α* coefficient exceeding 0.77 (Li et al., 2010). A threshold of ≥38 points has been established as optimal for clinical PTSD diagnosis in pregnant populations based on empirical evidence (Harrington and Newman, 2007).

*Cognitive Appraisal of Health Scale (CAHS)*. The CAHS originally developed by Kessler (1998), the Cronbach’s *α* coefficients for each dimension of the scale are all >0.70. The primary appraisal component comprises four domains: Threat, Challenge, Benign/Irrelevant, Harm/Loss. The validated Chinese version has been specifically adapted for pregnant populations (Tian et al., 2023), maintaining satisfactory reliability. This study utilized the 23-item primary appraisal section with a 5-point Likert scale. Higher scores indicate greater propensity to employ the corresponding cognitive appraisal pattern when confronting stressors.

*Trait Coping Style Questionnaire (TCSQ)*. The TCSQ developed by Chinese researchers (Jiang and Zhu, 1999), assesses individuals’ stress response patterns through two subscales: Positive Coping (PC) and Negative Coping (NC). Each subscale contains 10 items rated on a 5-point Likert scale, with higher scores indicating stronger tendencies toward the respective coping style. Evaluations demonstrate: Adequate internal consistency for the full scale (Cronbach’s *α* = 0.78). Satisfactory test–retest reliability: PC subscale *r* = 0.75, NC subscale *r* = 0.65.

*Perceived Social Support Scale (PSSS)*. The PSSS was developed by Zimet et al. (1990), consists of 12 items designed to assess support from family, friends, relatives, and coworkers. A 7-point Likert scale was used, with a total score ranging from 12 ~ 84, with higher scores indicating higher perceived social support. The Chinese version was revised by Wang et al. (1999), showed excellent reliability with Cronbach’s *α* of 0.99. In the pregnancy group, according to the validation Cronbach’s *α* was 0.89 (Liu et al., 2021), the PSSS was categorized into 3 dimensions, family, friends and other people in this study.

*Connor-Davidson Resilience Scale (CD-RISC)*. CD-RISC was originally developed by Connor and Davidson (2003) and consists of 25 items designed to measure an individual’s psychological resilience in response to stressful events. The 10-item simplified version of the CD-RISC was revised by Campbell-Sills and Stein (2007). A 5-point Likert scale was used, with a total score ranging from 0 ~ 40, with higher scores indicating higher levels of psychological resilience, i.e., greater resilience. Its Cronbach’s *α* was validated at 0.85 in trauma-exposed women, which was highly correlated with the original assessment tool in other domains (*r* = 0.92). The Chinese version of the CD-RISC-10 (Wang L. et al., 2010) had good internal consistency (Cronbach’s *α* = 0.91) and retest reliability (2-week *r* = 0.90).

#### 2.3.2 Analytic strategy

This study employed a two-step analysis method to integrate psychological variables with the trajectory modeling system: The first step used the repeated measurement scores of P-PTSD during the perinatal period at 4 time points as the modeling indicators. Through GBTM, the symptom trajectory subgroups were divided (i.e., low-risk stable group, medium-risk deteriorating group, high-risk fluctuating group). During this stage, cognitive evaluation, coping styles, social support, psychological resilience, etc. were not included as psychological variables to ensure that the trajectory classification was purely based on symptom change data. The second step used the 3 trajectory groups divided by GBTM as the multi-class dependent variables (with the low-risk stable group as the reference). Based on the stress system model, the repeated measurement values of the 4 psychological variables were used as the core predictive independent variables and included in the multi-class Logistic regression model. At the same time, demographic and obstetric-related confounding factors were controlled to analyze the independent predictive effect of each psychological indicator on the individual trajectory classification. This study did not directly embed psychological covariates into the GBTM model but adopted the standard two-step analysis framework of “first classification, then prediction of group attribution” to achieve the organic combination of theoretical psychological constructs and trajectory modeling.

#### 2.3.3 Theory—variable mapping

In the stress system model, the psychological processing of individuals when facing stressors involves four core components: cognitive evaluation, coping styles, social support, and psychological resilience. This study used the following tools to operationalize the above constructs: cognitive evaluation was measured using the Health-Cognitive Evaluation Scale (CAHS); coping styles were measured using the Trait Coping Style Questionnaire (TCSQ), which was divided into two sub-scales: positive coping and negative coping; social support was measured using the Perceived Social Support Scale (PSSS); and psychological resilience was measured using the Brief Version of the Coping Style Resilience Inventory (CD-RISC-10). The measurement indicators of these four constructs were all included as core predictor variables and were incorporated into the second step of the multinomial Logistic regression analysis.

### 2.4 Procedure

**Figure 3:**
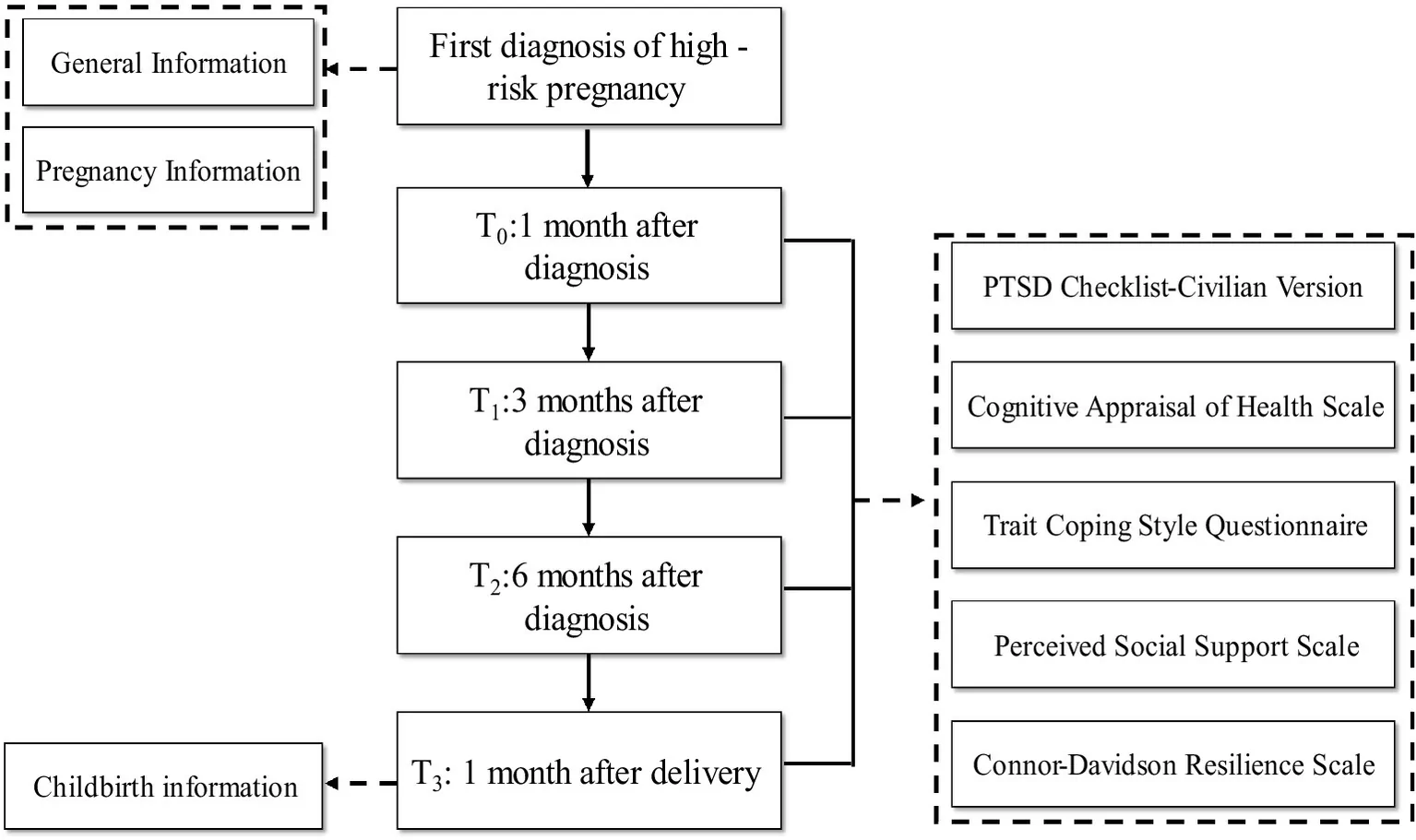
The schematic diagram of the timing and content of data collection.

Prior to the initiation of the study, all participants were provided with a comprehensive explanation of the study’s purpose and significance. Informed consent was obtained from all participants through the signature of the informed consent form. The study protocol was approved by the Institutional Review Board of Lanzhou University School of Nursing (approval number: LZUHLXY20230019). The schedule and content of data collection are illustrated in Figure 3.

### 2.5 Statistical analysis

The analysis of the data was conducted using SPSS 26.0 software. Data that exhibited a normal distribution were expressed as the mean ± standard deviation. Data that did not exhibit a normal distribution were expressed as the median and interquartile range. Count data were expressed as percentages. To assess changes in total P-PTSD scores and dimension scores across time points, generalized estimating equations (GEE) and repeated-measures ANOVA were employed. The analyses were executed using group trajectory modeling in Stata/MP17.0 software. GBTM is proposed to adopt the censored normal distribution (CNORM), which is applicable to the continuous PCL-C total score variable. The model selection follows the following four criteria: (1) lower Bayesian Information Criterion (BIC), Akaike Information Criterion (AIC), and sample-corrected BIC (aBIC) values; (2) entropy value ≥ 0.70, indicating acceptable classification accuracy; (3) each trajectory group should account for at least 5% of the total sample to ensure the stability of parameter estimation; (4) the clinical interpretability of the identified trajectories. Based on these criteria, we tested models with 2 to 5 trajectory groups and different polynomial orders (linear, quadratic term, cubic term) combinations. Finally, the model with the optimal fit and the most clinical significance was selected. The polynomial order (such as 1, 2, 2) of the final model was determined based on the lowest BIC and comprehensive consideration of clinical interpretability. The t-test and χ^2^ test were used to explore the influencing factors of different types of P-PTSD trajectories, and a difference of *p* < 0.05 was considered statistically significant.

*Sample size adequacy for trajectory analyses*. We completed *a priori* sample size calculation for estimating the overall P-PTSD prevalence before participant enrollment, while dedicated pre-study power simulation specifically for GBTM and multinomial logistic regression was not conducted given the exploratory longitudinal design of this work. We therefore retrospectively verified sample adequacy via established methodological criteria: For group-based trajectory modeling (GBTM): Nagin (2005) recommended 30–50 participants per trajectory subgroup to guarantee stable parameter estimation. Our smallest high-fluctuation trajectory group contained 42 participants (17.1% of the analytical sample), which falls within this recommended range. Moreover, the entropy value of 0.71 (Table 1) reflected acceptable subgroup classification accuracy, further supporting the reliability of our three-trajectory model solution. For multinomial logistic regression with multiple predictors: To prevent model overfitting within the limited size of the smallest subgroup, we adopted a parsimonious variable selection strategy. Only theoretical core psychological constructs and covariates with significant univariate associations were retained in the final regression model.

**Table 1:** P-PTSD symptomatology alternative developmental pathways potential category fit indicators.

Categories	*LL*	*BIC*	*aBIC*	*AIC*	*Entropy*	%				
1(3)	−3473.92	−3491.14	−3487.67	−3478.92	0.00	100.0				
2(3 3)	−3414.75	−3449.18	−3442.25	−3424.75	0.63	62.3	37.7			
2(2 2)	−3418.30	−3445.85	−3440.30	−3426.30	0.61	59.4	40.6			
3(3 3 3)	−3394.40	−3446.06	−3435.66	−3409.40	0.72	37.2	45.9	16.9		
3(2 2 2)	−3399.53	−3440.86	−3432.54	−3411.53	0.72	35.4	48.4	16.2		
3(1 2 2)	**−3400.19**	**−3438.08**	**−3430.45**	**−3411.19**	**0.71**	**36.1**	**46.8**	**17.1**		
4(3 3 3 3)	−3383.37	−3452.25	−3438.39	−3403.37	0.71	43.3	12.1	40.7	3.9	
4(2 2 2 2)	−3389.70	−3444.80	−3433.71	−3405.70	0.70	40.9	11.1	43.5	4.5	
5(3 3 3 3 3)	−3376.58	−3462.67	−3445.34	−3401.58	0.67	25.6	40.2	10.1	23.1	1.0

The bold values in Table indicate the optimal model selected based on the following criteria: (1) the smallest Bayesian Information Criterion (BIC), sample-size adjusted BIC (aBIC), and Akaike Information Criterion (AIC); (2) an Entropy value ≥ 0.70, indicating acceptable classification accuracy; (3) the proportion of each trajectory group ≥ 5% of the total sample to ensure stable parameter estimation. Based on these criteria, the 3-group model with polynomial orders (1, 2, 2) was selected as the final model.

## 3 Results

### 3.1 Sample characteristics

**Figure 4:**
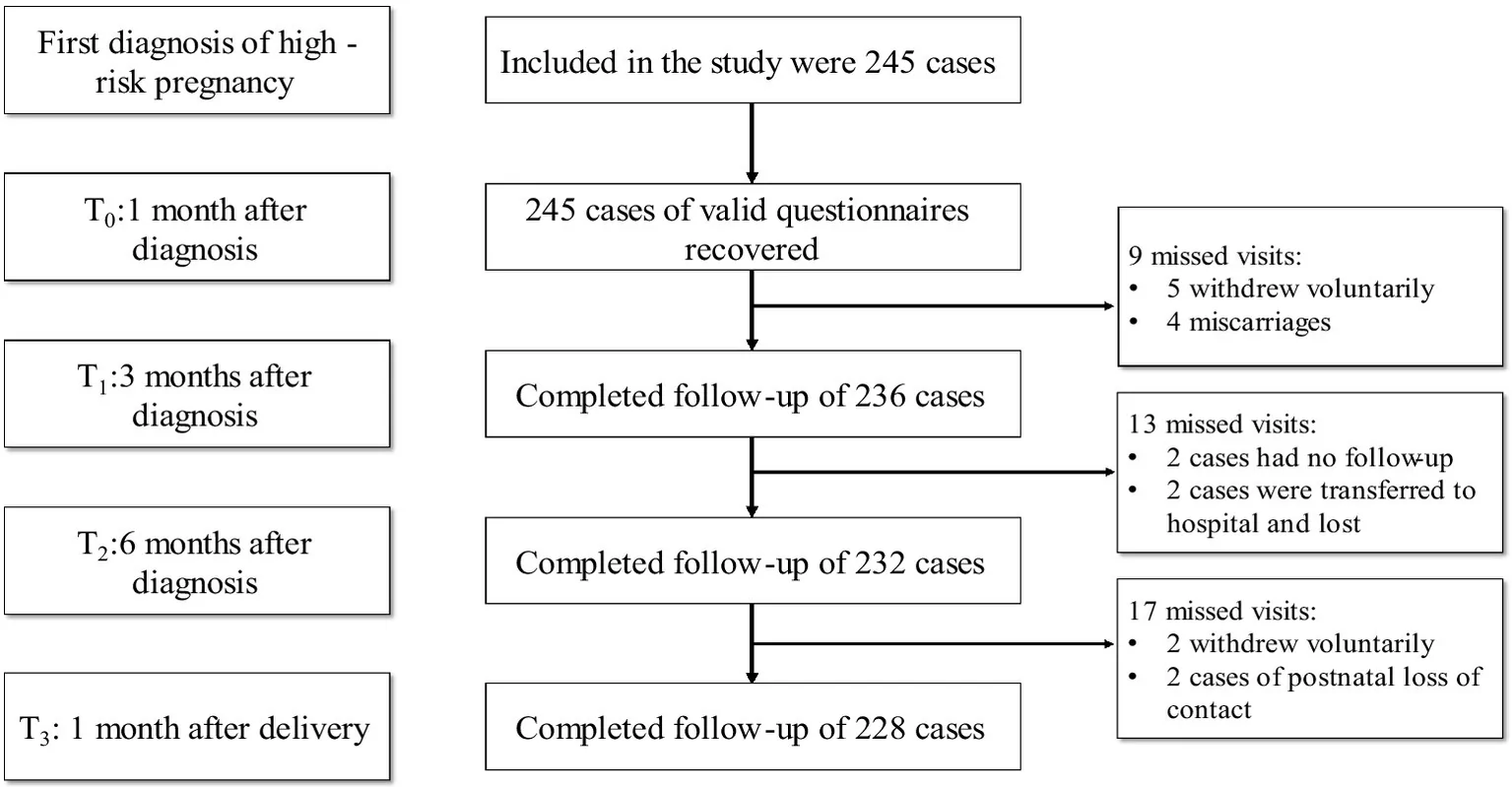
Perinatal follow-up flowchart.

Baseline data were collected from February to May of 2023, and perinatal follow-up was conducted until December of 2023. The perinatal follow-up workflow is illustrated in Figure 4.

### 3.2 Baseline characteristics of the study population

A total of 245 high-risk pregnant women were included in the study. By comparing the demographic and pregnancy-related data of 228 cases of high-risk pregnant women who completed the perinatal follow-up with 17 cases of lost visits, the difference in the distribution of relevant indicators was not statistically significant after testing (all *p* > 0.05). Further calculation of effect size indicates that the Cohen’s d for the continuous variable (age) is 0.004, which is far below the small effect threshold (d = 0.20); the Cramer’s V values for the categorical variables range from 0.002 to 0.151, with only “the number of high-risk factors” (V = 0.151) slightly exceeding 0.10, and the rest are all below the small effect threshold. The combination of low dropout rate (6.9%), no statistical difference, and small effect size suggests that the dropout bias is unlikely to have a substantial impact on the results of this study. See Tables 2, 3 for details.

**Table 2:** Analyzing the general population and lost visit information.

Item	Category	Subjects included 245 cases		Perinatal follow-up completed 228 cases		Lost to interview 17 cases		P*	Effect sizes
		*N*	*%*	*N*	*%*	*N*	*%*		
Demographics									
Age (years)	18 ~ 29	85	34.7	78	34.2	7	41.2	0.624	Cohen‘s d = 0.004
	30 ~ 34	111	45.3	103	45.2	8	47.1		
	≥35	49	20.0	47	20.6	2	11.8		
Household register	Agriculture	59	24.1	57	25.0	2	11.8	.349^b^	
	Non-agriculture	186	75.9	171	75.0	15	88.2		
One-child	Yes	216	88.2	199	87.3	17	100.0	.239^b^	
	No	29	11.8	29	12.7	/	/		
Ethnicity	Han	231	94.3	215	94.3	16	94.1	.999^b^	Cramer’s V = 0.002
	Other	14	5.7	13	5.7	1	5.9		
Religious belief	Yes	13	5.3	13	5.7	/	/	.652^b^	Cramer’s V = 0.065
	No	232	94.7	215	94.3	17	100.0		
Education level	Lower secondary and below	25	10.2	23	10.1	2	11.8	0.644	
	High school	34	13.9	33	14.5	1	5.9		
	Tertiary	75	30.6	70	30.7	5	29.4		
	Undergraduate	90	36.7	82	36.0	8	47.1		
	Postgraduate and above	21	8.6	20	8.8	1	5.9		
Monthly household income(yuan)	<3,000	28	11.4	27	11.8	1	5.9	0.495	
	3,001 ~ 6,000	103	42.0	93	40.8	10	58.8		
	6,001 ~ 9,000	67	27.3	63	27.6	4	23.5		
	>9,000	47	19.2	45	19.7	2	11.8		
Personal work	Government enterprises/agencies	69	28.2	66	28.9	3	17.6	0.443	
	Private company	54	22.0	47	20.6	7	41.2		
	Individual/farmer	44	18.0	41	18.0	3	17.6		
	No work	36	14.7	34	14.9	2	11.8		
	Otherwise	42	17.1	40	17.5	2	11.8		
Marital status	First marriage	228	93.1	213	93.4	15	88.2	.751^b^	
	Remarriage	17	6.9	15	6.6	2	11.8		
Family structure	Two-person family	158	64.5	146	64.0	12	70.6	.778^b^	Cramer’s V = 0.035
	Living with parents	87	35.5	82	36.0	5	29.4		
Medical payment methods	Provincial/Employee health insurance	31	12.7	29	12.7	2	11.8	0.654	
	Municipal health insurance	75	30.6	71	31.3	4	23.5		
	Resident/ Rural cooperative medical care	55	22.4	49	21.5	6	35.3		
	Self-financed	84	34.3	79	34.6	5	29.4		
Personality traits(self-evaluation)	Introversion	39	15.9	35	15.4	4	23.5	0.683	
	Extraversion	69	28.2	65	28.5	4	23.5		
	Mesomorphic	137	55.9	128	56.1	9	52.9		
Experiencing severe trauma	Yes	126	51.4	116	50.9	10	58.8	0.703^b^	Cramer’s V = 0.040
	No	119	48.6	112	49.1	7	41.2		
Pregnancy related information									
Marriage and childbearing interval (months)	0 ~ 6	98	40.0	89	39.0	9	52.9	0.852	
	7 ~ 12	72	29.4	67	29.4	5	29.4		
	13 ~ 24	37	15.1	35	15.4	2	11.8		
	≥25	38	15.5	37	16.2	1	5.9		
History of abortion	0	127	51.8	117	51.3	10	58.8	0.547	Cramer’s V = 0.071
	1	86	35.1	80	35.1	6	35.3		
	2	19	7.8	18	7.9	1	5.9		
	≥3	13	5.3	13	5.7	/	/		
Causes of abortion (history of abortion)	Spontaneous abortion	39	28.5	34	26.8	2	25.0	0.829	
	Providing abortion	92	67.2	87	68.5	5	62.5		
	Medication abortion	6	4.3	6	4.7	1	12.5		
Whether the pregnancy was planned	Planned	138	56.3	128	56.1	10	58.8	0.479^b^	Cramer’s V = 0.014
	Unplanned	107	43.7	100	43.9	7	41.2		
Pregnancy method (current pregnancy)	Natural Pregnancy	209	85.3	193	84.6	16	94.1	0.809^b^	Cramer’s V = 0.068
	Assisted Reproduction	36	14.7	35	15.4	1	5.9		
Number of livebirths	First	144	58.8	139	61.0	5	29.4	0.032	
	Second	83	33.9	74	32.5	9	52.9		
	≥ Third	18	7.3	15	6.6	3	17.6		
Fetus	Single Pregnancy	227	92.7	212	93.0	15	88.2	0.809	
	Multiple Pregnancy	18	7.3	16	7.0	2	11.8		
Pregnancy risks	Yellow(general risk)	111	45.3	98	43.0	13	76.5	0.063	
	Orange (medium risk)	115	46.9	112	49.1	3	17.6		
	Purple (infectious diseases)/ Combined with other colours	19	7.8	18	7.9	1	5.9		
Number of high-risk factors	1	98	40.0	87	38.2	11	64.7	0.087	Cramer’s V = 0.151
	2	88	35.9	83	36.4	5	29.4		
	≥3	59	24.1	58	25.4	1	5.9		

Expected counts <5 in cells for the lost visit population were calculated using the correction formula; the 2 × 2 table was calculated using the continuity-corrected b-test.

**Table 3:** Delivery status of research subjects.

Item	Category	*N*	*%*
Breast feeding	No	28	12.3
	Mixed feeding	95	41.7
	Yes	105	46.1
Labour analgesia	No	109	47.8
	Pharmacological analgesia	88	38.6
	Non-pharmacological analgesia	31	13.6
Delivery mode	Natural	96	42.1
	Instrumentally assisted childbirth	22	9.6
	Planned caesarean section	77	33.8
	Negative caesarean section	15	6.6
	Emergency caesarean section	18	7.9
Complications of childbirth	No	178	78.1
	Postpartum hemorrhage	11	4.8
	Puerperal infection	19	8.3
	Acute mastitis	5	2.2
	Urinary system infection	15	6.6
Situation of newborns	Healthy/good	161	70.6
	Greater than/less than gestational age	18	7.9
	High/low birth weight	12	5.3
	NICU hospitalization	37	16.2
Number of neonatal complications	0	90	39.5
	1	101	44.3
	≥2	37	16.2
Neonatal complications	No	90	33.3
	Neonatal jaundice	98	36.3
	Gluteal red	15	5.6
	Umbilical infection	8	3.0
	Neonatal diarrhea	21	7.8
	Vomiting	9	3.3
	Neonatal fever	13	4.8
	Seizure	16	5.9
Fetal gender expectations	Meet expectations	215	94.3
	Does not meet expectations	13	5.7

### 3.3 The course of P-PTSD in women at high risk during pregnancy

#### 3.3.1 General trends

The positive detection rates of P-PTSD at T_0_-T_3_ were 12.7, 29.8, 31.4, and 35.9%. Generalised estimating equations were used to analyse the dynamic changes in P-PTSD from T_0_ to T_3_. The results showed that the differences in the total P-PTSD scores and scores for each dimension among the 245 high-risk pregnant women were statistically significant, indicating that the symptoms of post-traumatic stress disorder in the study subjects showed an overall upward trend., as shown in Table 4.

**Table 4:** Changes in P-PTSD total score and dimension scores.

Variables/dimensions	1 month after diagnosis	3 months after diagnosis	6 months after diagnosis	1 month after delivery	*χ* ^2^	P*
	T_0_	T_1_	T_2_	T_3_		
P-PTSD total score	23.0(19.0 ~ 30.0)	24.0(19.0 ~ 38.0)	27.5(20.0 ~ 39.0)	32.0(22.0 ~ 39.0)	64.02	0.001
Re-experiencing	6.0(5.0 ~ 8.0)	7.0(5.0 ~ 10.8)	7.5(5.0 ~ 11.0)	9.0(7.0 ~ 11.5)	71.31	0.001
Avoidance/numbing	8.0(7.0 ~ 11.0)	9.0(7.0 ~ 13.0)	10(7.0 ~ 15.0)	12.0(8.0 ~ 16.0)	59.26	0.001
High alertness	8.0(6.0 ~ 10.0)	8.5(6.0 ~ 12.8)	9.0(7.0 ~ 12.0)	10.0(7.0 ~ 13.0)	24.58	0.001

#### 3.3.2 Possible categories and characteristics

The group trajectory model GBTM was used to fit the perinatal P-PTSD from T_0_ to T_3_. The initial fit starts from the higher order term, comparing the trajectory model indicators of each category, when 3 categories are reached, the absolute values of Log likelihood(*LL*), Bayesian information criterion(*BIC*), Sample size adjusted Bayesian information criterion(*aBIC*) and Akaike information criterion(*AIC*)are the smallest, and the Entropy Accuracy Index is acceptable, see Table 1.

Meanwhile, the intercept, linear and quadratic parameters of each group of trajectories fitted by combining appropriate combinations of higher order terms of the 3 types of trajectories are shown in Table 5.

**Table 5:** Evaluation of the effectiveness of the fitted metrics within the Group Trajectory Model.

Categories	Parameter	β	SE	t	P*
Category 1	Intercept	19.68	1.09	18.05	<0.001
	Linear	0.45	0.17	2.62	<0.01
Category 2	Intercept	26.96	0.98	27.56	<0.001
	Linear	1.71	0.47	3.66	0.001
	Square	−0.08	0.05	−1.68	0.09
Category 3	Intercept	40.62	3.77	10.77	<0.001
	Linear	3.32	1.66	2.00	<0.05
	Square	−0.26	0.17	−1.54	0.12

**Figure 5:**
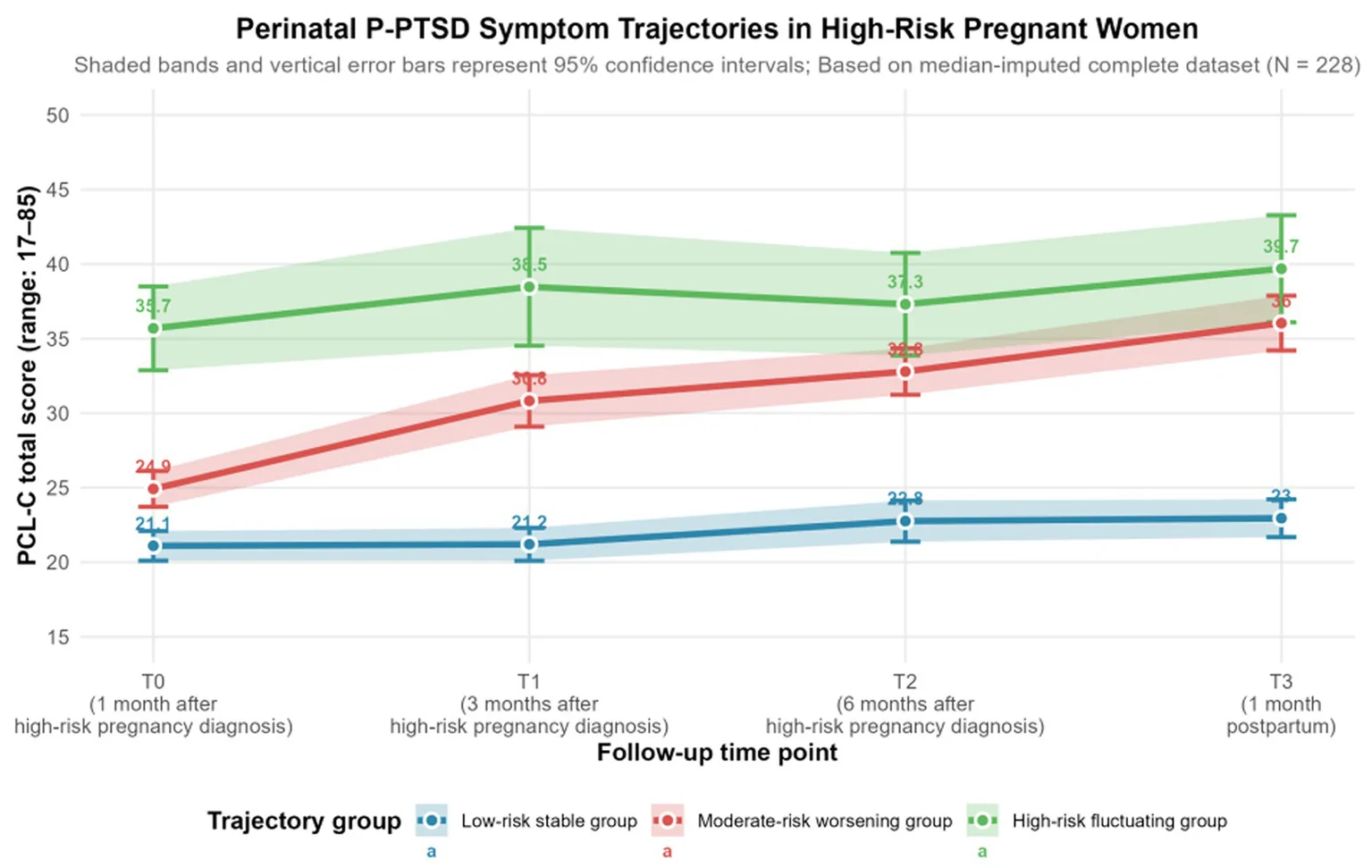
Trajectory of change in P-PTSD symptoms.

The fitted model trajectories of the potential categories in the perinatal P-PTSD total scores T_0_-T_3_ can correspond to 3 distinctly different, significantly different change curves, as shown in Figure 5. The trajectory models were named according to their respective characteristics: *Category 1* (88 cases, 36.1%, blue curve) showed that the study participants’ PCL-C scale scores were at a low level of stabilization, indicating that P-PTSD symptoms were mild and persisted until final follow-up, thus it was named as the low-risk stable group. *Category 2* (115 cases, 46.8%, red curve) showed that the PCL-C scale scores of the study participants were higher in the T_0_–T_3_ phase than in the first category, with mean values of the intercept and slope of 26.96 and 1.71 (*p* < 0.001), respectively, indicating an overall upward trend. Therefore, it was named as the moderate-risk worsening group. *Category 3* (42 cases, 17.1%, green curve) showed that during the perinatal period (T_0_-T_3_), the PCL-C scale scores of the study participants remained high and fluctuated, first increasing, then decreasing, and then increasing again. This group of mothers exhibited higher P-PTSD symptoms in the month following the diagnosis of a high-risk pregnancy and in the first postpartum month. Hence, it was named as the high-risk fluctuating group.

### 3.4 Factors influencing the P-PTSD trajectory model for high-risk pregnant women

#### 3.4.1 Results of single factor analysis

The results showed that family type, education, time between first marriage and first birth, history of spontaneous abortion, delivery complications, neonatal health status, number of complications, and gender predicted changes in the developmental trajectory of P-PTSD in high-risk pregnant women (*p* < 0.05), as shown in Table 6.

**Table 6:** Univariate analysis of different P-PTSD trajectory types.

Item	The low-risk stable group (88)		The moderate-risk worsening group (115)		The high-risk fluctuating group (42)		*χ^2^*	P*
	*N*	%	*N*	%	*N*	%		
Demographics								
Age (years)							8.39	0.078
18 ~ 29	32	36.3	45	39.1	8	19.0		
30 ~ 34	43	48.9	44	38.3	24	57.2		
≥35	13	14.8	26	22.6	10	23.8		
Household register							61.45	0.001
Agriculture	6	6.8	24	20.9	29	69.0		
Non-agriculture	82	93.2	91	79.1	13	31.0		
One-Child							3.02	0.221
Yes	11	12.5	16	13.9	2	4.8		
No	77	87.5	99	86.1	40	95.2		
Ethnicity							1.41	0.495
Han	83	94.3	107	93.0	41	95.2		
Other	5	5.7	8	7.0	1	2.4		
Religious beliefs							2.86	0.240
Yes	2	2.3	8	7.0	3	7.1		
No	86	97.7	107	93.0	39	92.9		
Education level							20.25	0.009
Lower secondary and below	6	6.8	12	10.4	7	16.6		
High school	7	8.0	14	12.1	13	31.0		
Tertiary	30	34.1	37	32.2	8	19.0		
Undergraduate	33	37.5	44	38.3	13	31.0		
Postgraduate and above	12	13.6	8	7.0	1	2.4		
Monthly household income(yuan)							15.98	0.014
< 3,000	4	4.6	18	15.6	6	14.3		
3,001 ~ 6,000	31	35.2	52	45.2	20	47.6		
6,001 ~ 9,000	31	35.2	24	20.9	12	28.6		
> 9,000	22	25.0	21	18.3	4	9.5		
Personal work							8.87	0.354
Government enterprises/agencies	25	28.4	31	26.9	13	30.9		
Private company	23	26.1	24	20.9	7	16.7		
Individual/farmer	20	22.7	17	14.8	7	16.7		
No work	12	13.7	17	14.8	7	16.7		
Otherwise	8	9.1	26	22.6	8	19.0		
Marital status							1.70	0.427
First marriage	83	94.3	108	93.9	37	88.1		
Remarriage	5	5.7	7	6.1	5	11.9		
Family structures							1.21	0.547
Two-person family	53	60.2	76	66.1	29	69.0		
Living with parents	35	39.8	39	33.9	13	31.0		
Medical payment methods							4.86	0.562
Provincial/employee health insurance	14	15.9	12	10.4	5	11.9		
Municipal health insurance	27	30.7	32	27.8	16	38.1		
Resident/rural cooperative medical care	18	20.4	26	22.6	11	26.2		
Self-financed	29	33.0	45	39.2	10	23.8		
Personality traits(self-evaluation)							9.74	0.045
Introversion	9	10.2	18	15.7	12	28.6		
Extraversion	29	33.0	34	29.5	6	14.3		
Mesomorphic	50	56.8	63	54.8	24	57.1		
Experiencing severe trauma							24.17	0.001
No	47	53.4	66	57.4	6	14.3		
Yes	41	46.6	49	42.6	36	85.7		
Pregnancy related information								
Marriage and childbearing interval (months)							20.00	0.003
0 ~ 6	40	45.4	49	42.6	9	21.4		
7 ~ 12	31	35.2	31	26.9	10	23.8		
13 ~ 24	10	11.4	18	15.7	9	21.4		
≥25	7	8.0	17	14.8	14	33.4		
History of abortion							4.92	0.555
0	42	47.7	63	54.8	22	52.4		
1	36	40.9	38	33.0	12	28.6		
2	5	5.7	8	7.0	6	14.3		
≥3	5	5.7	6	5.2	2	4.7		
Causes of abortion(history of abortion)								
Spontaneous abortion	17	19.3	11	9.6	11	26.2	7.54	0.023
Labor abortion	35	39.8	45	39.1	12	28.6	1.75	0.417
Medical abortion	2	2.3	5	4.3	/	/	2.26	0.322
Whether the pregnancy was planned							29.17	0.001
Planned	65	73.9	63	54.8	10	23.8		
Unplanned	23	26.1	52	45.2	32	76.2		
Pregnancy method(current pregnancy)							1.22	0.545
Natural pregnancy	78	88.6	96	83.5	35	83.3		
Assisted reproduction	10	11.4	19	16.5	7	16.7		
Number of livebirths							4.09	0.395
First	56	63.6	67	58.3	21	50.0		
Second	25	28.4	42	36.5	16	38.1		
≥ Third	7	8.0	6	5.2	5	11.9		
Fetus							6.19	0.045
Single pregnancy	86	97.7	103	89.6	38	90.5		
Multiple pregnancy	2	2.3	12	10.4	4	9.5		
Pregnancy risks							6.24	0.182
Yellow(general risk)	49	55.7	57	49.6	17	40.5		
Orange(medium risk)	35	39.8	50	43.5	18	42.8		
Purple(infectious diseases)/ Combined with other colours	4	4.5	8	6.9	7	16.7		
Number of high-risk factors							8.57	0.073
1	45	51.1	41	35.6	12	28.6		
2	25	28.4	43	37.4	20	47.6		
≥3	18	20.5	31	27.0	10	23.8		
Information on childbirth								
Breast feeding							21.38	0.001
No	4	4.7	16	15.4	8	20.5		
Mixed feeding	51	60.0	33	31.7	11	28.2		
Yes	30	35.3	55	52.9	20	51.3		
Labour analgesia							15.65	0.004
No	27	31.8	59	56.7	23	59.0		
Pharmacological analgesia	40	47.0	35	33.7	13	33.3		
Non-pharmacological analgesia	18	21.2	10	9.6	3	7.7		
Delivery mode							14.24	0.001
Natural/instrumentally assisted childbirth	56	65.9	50	48.1	12	30.8		
Planned/emergency/negative caesarean section	29	34.1	54	51.9	27	69.2		
Complications of childbirth							24.73	0.001
No	80	94.1	75	72.1	23	59.0		
Yes	5	5.9	29	27.9	16	41.0		
Situation of newborns							39.51	0.001
Healthy/good	78	91.8	68	65.4	15	38.5		
Otherwise	7	8.2	36	34.6	24	61.5		
Number of neonatal complications							27.53	0.001
0	49	57.6	31	29.8	10	25.6		
1	32	37.7	48	46.2	21	53.9		
≥2	4	4.7	25	24.0	8	20.5		
Fetal gender expectations							6.65	0.036
Meet expectations	84	98.8	96	92.3	35	89.7		
Does not meet expectations	1	1.2	8	7.7	4	10.3		

Repeated measures ANOVA was used to further validate the differences in core psychological distress variables among high-risk pregnant women with different P-PTSD trajectories at T_0_–T_3_, the results were shown in Table 7.

**Table 7:** Repeated-measures ANOVA of core psychological stress variables for different P-PTSD trajectory types.

Variables/dimensions ( $\bar{x}$ ±S)	Time	The low-risk stable group	The moderate-risk worsening group	The high-risk fluctuating group	Time main effect (P*)	Group main effect (P*)	Interactive effect (P*)
		*N* = 88	*N* = 115	*N* = 42			
Positive appraisal	T_0_	39.02 ± 6.08	40.39 ± 5.13	40.05 ± 5.08	<0.001	<0.001	<0.001
	T_1_	37.00 ± 4.68	34.46 ± 4.85	32.29 ± 4.52			
	T_2_	36.07 ± 4.22	33.52 ± 5.33	32.71 ± 5.13			
	T_3_	36.35 ± 4.39	32.34 ± 4.75	31.29 ± 5.55			
Negative appraisal	T_0_	25.62 ± 8.35	28.53 ± 7.57	31.05 ± 7.73	<0.001	<0.001	<0.001
	T_1_	27.02 ± 7.10	31.27 ± 8.60	35.79 ± 7.93			
	T_2_	27.11 ± 6.82	32.05 ± 8.66	32.36 ± 8.00			
	T_3_	27.00 ± 6.50	35.80 ± 8.92	36.60 ± 8.51			
Negative coping	T_0_	20.70 ± 5.67	23.69 ± 6.60	24.55 ± 5.42	<0.001	<0.001	0.390
	T_1_	22.99 ± 6.82	26.73 ± 6.73	29.48 ± 5.65			
	T_2_	23.11 ± 5.97	26.95 ± 6.48	28.00 ± 6.88			
	T_3_	24.14 ± 7.51	28.35 ± 6.12	27.95 ± 4.49			
Positive coping	T_0_	35.18 ± 6.34	31.17 ± 8.18	30.93 ± 5.67	0.830	<0.001	0.610
	T_1_	34.72 ± 5.86	31.83 ± 6.11	29.26 ± 4.82			
	T_2_	34.14 ± 5.12	31.79 ± 5.29	30.60 ± 4.72			
	T_3_	34.51 ± 5.35	31.43 ± 4.74	30.00 ± 5.66			
Social support	T_0_	64.69 ± 11.82	62.62 ± 11.82	57.71 ± 9.18	0.020	<0.001	0.090
	T_1_	62.51 ± 12.29	59.30 ± 11.65	54.00 ± 10.44			
	T_2_	62.90 ± 11.47	59.39 ± 10.99	55.57 ± 11.54			
	T_3_	66.23 ± 11.09	57.77 ± 11.60	55.17 ± 9.71			
Psychological resilience	T_0_	38.74 ± 5.57	36.66 ± 7.06	34.26 ± 5.70	<0.010	<0.001	0.360
	T_1_	38.16 ± 5.54	34.63 ± 6.70	32.98 ± 5.09			
	T_2_	38.22 ± 6.34	33.90 ± 6.14	32.33 ± 5.71			
	T_3_	38.26 ± 5.65	33.50 ± 6.70	31.45 ± 6.58			

T_0_ to T_3_ are 1, 3, 6 and 1 month postpartum after diagnosis of high-risk pregnancy, respectively.

#### 3.4.2 Multifactor logistic regression analysis

The three P-PTSD developmental trajectory types were utilized as dependent variables, and statistically significant factors from the univariate analysis were selected as predictor variables to be incorporated into the regression model. Logistic regression analyses were conducted by incorporating the pertinent variables at the test level of *α*_inclusion_ = 0.05 and *β*_exclusion_ = 0.10. The findings of the multicategorical logistic regression model are presented in Table 8.

**Table 8:** Unordered multicategorical regression analysis of P-PTSD symptom course predictors.

Categories	Predictor variable	Reference variable(the low-risk stable group)	*B*	*Wald*	P*	*OR*	95%*CI*	
							Lower limits	Upper limits
The medium-risk deterioration group^a^	T_1_ positive coping	T_1_ positive coping	−0.14	4.22	0.040	0.87	0.76	0.99
	T_2_ negative coping	T_2_ negative coping	0.11	5.13	0.024	1.11	1.02	1.22
	T_3_ negative appraisal	T_3_ negative appraisal	0.18	12.04	0.001	1.19	1.08	1.32
	no neonatal complication	Neonatal complications ≥ 2 and only 1 neonatal complication	−2.42	4.90	0.027	0.09	0.01	0.76
	only 1 neonatal complication	Neonatal complications ≥ 2 and no neonatal complication	−2.46	5.60	0.018	0.09	0.01	0.66
	average monthly household income of 6,001~9000yuan	Average monthly household income:<3,000, 3,001 ~ 6,000 and >9,000	−1.69	4.62	0.032	0.19	0.04	0.86
The high-risk fluctuation group ^a^	T_1_ negative coping	T_1_ negative coping	0.20	7.74	0.005	1.22	1.06	1.40
	T_1_ positive coping	T_1_ positive coping	−0.23	5.53	0.019	0.79	0.66	0.96
	T_2_ negative coping	T_2_ negative coping	0.24	10.64	0.001	1.27	1.10	1.46
	T_3_ negative appraisal	T_3_ negative appraisal	0.19	9.96	0.002	1.21	1.08	1.36
	Previous non-traumatic experience	Previous traumatic experience	−1.80	4.17	0.041	0.17	0.03	0.93
	Natural/device-assisted birth	Planned/emergency/negative caesarean section	−1.79	3.66	0.046	0.17	0.03	1.04

a The low-risk stable group used as reference group.

## 4 Discussion

### 4.1 Longitudinal trajectory of P-PTSD in high-risk perinatal women

A comparative analysis was conducted of the incidence of P-PTSD in high-risk pregnant women, utilising data from previous studies. The prevalence of P-PTSD in high-risk pregnant women with gestational ages ≥28 weeks in China was found to be 15.40% (Huang, 2019). International studies have demonstrated that the incidence of P-PTSD in high-risk pregnant women with gestational ages ≥28 weeks can reach up to 18.95% (Yildiz et al., 2017). However, no studies have been identified that specifically address the issue of high-risk pregnant women in the postpartum period. The incidence of non-specific group positivity ranged from 9.52% ~ 12.12% and was mostly screened at 42 days postpartum, with a higher incidence in women with cesarean section, preterm labor, NICU, etc. (17.55% ~ 39.5%) (Williams et al., 2023; Chen, 2021; Zheng et al., 2022), and as high as 42.3% in some studies (Rawal et al., 2017). Therefore, the relatively high positive detection rate of T_1_ and T_2_ time points in this study suggests that P-PTSD symptoms have a more serious impact on high-risk pregnant women in the middle and late stages of pregnancy and may represent a greater threat to mental health. And T_3_ had the highest positive detection rate, indicating that 1 month postpartum is the most likely time point for P-PTSD symptoms. This may be explained by the continuous exposure to the novel stressor of ‘childbirth’. Within a short period after delivery, mothers may not have fully adapted to the maternal role, which may be reflected in elevated levels of trauma re-experiencing, avoidance/numbing, and hyperarousal. This suggests that “childbirth” may have a greater impact on the maternal trauma experience, and that the focus should be on the later stages of pregnancy and the early postpartum period.

### 4.2 Trajectory subtypes and their clinical profiles

This study showed heterogeneity in the change of maternal P-PTSD symptoms with different characteristics in the same HRP state and identified trajectories in three categories. The three trajectory groups—low-risk stable, moderate-risk worsening, and high-risk fluctuating—reflect differential patterns of psychological resource utilization, consistent with recent findings that maternal mental health outcomes under sustained stress are shaped by individual coping resources and social support. The low-risk stable group: from a positive psychology perspective, some individuals undergo a series of complex cognitive processes following a stressful event, ultimately achieving positive psychological adaptation, known as post-traumatic growth (PTG) (Tedeschi et al., 2017). Research indicates (Berman et al., 2021; Chen et al., 2023; Sawyer and Ayers, 2009) that PTG is associated with lower levels of P-PTSD symptoms. The pregnant women in this group exhibited relatively low levels of PTSD symptoms when facing the stressful event of being diagnosed with a “high-risk pregnancy” suggesting positive psychological adaptation, improved behavioral adaptation, and enhanced social functioning, including new possibilities, life insights, closer relationships with others, increased personal coping abilities, and personal transformation (Wang J. et al., 2010). The medium-risk deterioration group: a study on the trajectory of P-PTSD development from 28 weeks of gestation to 6 weeks postpartum showed (Xu and Zhu, 2023) that the majority of pregnant women (58%) had higher symptom scores in mid- and late-gestation, with an increasing trend in the development of P-PTSD throughout the perinatal period (low, *p* = 0.03; decreasing, *p* < 0.001), which is in line with the results of this study. High pregnancy stress and exposure to trauma were significantly associated with the increased symptom burden in this group of pregnant women. Research indicates (Huang, 2019) that pregnancy stress has a positive direct effect on P-PTSD in high-risk pregnant women, with stressors primarily related to “ensuring the health and safety of mother and child.” The health threats posed by ‘high-risk pregnancy’ were associated with elevated pregnancy stress; prolonged exposure to such stress may, in turn, be related to adverse delivery outcomes and severe stress responses, which may further correlate with the development of P-PTSD. The high-risk fluctuation group: the study by Onoye et al. (2013) is in general agreement with the findings of this study. However, their trajectory showed that scores for P-PTSD increased significantly in late pregnancy. Whereas, in the present study, the highest trajectory point for this group was identified as mid-pregnancy, which may reflect changes in psychological stress responses during the third trimester of pregnancy. In addition, in the study by Onoye et al. there was a significant decrease in P-PTSD scores in the early postpartum period compared to the later stages of pregnancy. Considering the earlier screening time point in this study, and the fact that some socio-cultural factors contribute to higher levels of psychological stress, P-PTSD scores may have rebounded. Other longitudinal studies (Söderquist et al., 2006) have shown that P-PTSD does not decline over time between 1 and 11 months postpartum.

### 4.3 Analysis of contributing factors

#### 4.3.1 Sociodemographic characteristics

Multivariate logistic analysis indicated that, after controlling for other variables, high-risk pregnant women with P-PTSD who had a monthly household income of 6,001–9,000 yuan had a 0.19-fold reduction in the odds ratio (*OR* = 0.19, 95% *CI* = 0.04–0.86, *p* = 0.03) of developing into the moderate-risk deterioration group compared to the low-risk stable group. It is currently widely recognised that higher household income is a protective factor against P-PTSD, and household registration type can also indirectly reflect a certain level of economic and medical status (Craemer et al., 2023). In Cohen et al. (2004) study, pregnant women with household incomes at or above the median had a lower risk of developing P-PTSD symptoms (*OR* = 0.4, 95% *CI* = 0.2–0.8); when such income is secured, pregnant women and their families perceive lower risks, which facilitates emotional management and healthy decision-making. Educational attainment may also act as a limiting factor. Individuals with a bachelor’s degree or lower have a narrower knowledge base and may have only a superficial understanding of perinatal health knowledge, which may be related to psychological anxiety and unnecessary concerns. As labour progresses, they may exhibit a “risk escalation” state. In contrast, individuals with higher educational attainment, while having access to high-quality medical resources, demonstrate a deeper understanding of disease-related knowledge and maintain a low-risk, stable emotional state.

Additionally, after controlling for other variables, high-risk pregnant women who had not experienced major trauma had a 0.17-fold reduction in the odds ratio (*OR* = 0.17, 95% *CI* = 0.03 to 0.93, *p* = 0.04) of developing P-PTSD and being classified into the high-risk fluctuating group compared to the low-risk stable group. High-risk factors and childbirth may serve as traumatic events, triggering memories of past painful experiences such as childhood abuse, car accidents, or fires, and may therefore be associated with cumulative stress responses and elevated P-PTSD symptoms (Yampolsky et al., 2010). Goutaudier et al. (2014) also confirmed the predictive role of past traumatic events on P-PTSD.

#### 4.3.2 Information on pregnancy and childbirth

After controlling for other variables, Natural/assisted delivery was associated with a reduced risk of P-PTSD compared to the high-risk fluctuation group (compared to the low-risk stability group) (OR = 0.17, 95% CI = 0.03–1.04, *p* = 0.046), but the upper limit of the confidence interval was close to 1.0, suggesting that this association was marginally significant and requires further verification in future studies; For high-risk pregnant women with no or only one neonatal complication, the odds ratio for P-PTSD developing into the moderate-risk deterioration group compared to the low-risk stable group was reduced by 0.09 (*OR* = 0.09, *p* < 0.05). The predictive value of these two protective factors for P-PTSD development has also been confirmed in other studies (Steetskamp et al., 2022). For high-risk pregnant women (Almeida et al., 2023), complex pregnancy conditions are associated with higher rates of neonatal complications and caesarean sections, which may contribute to greater short- and long-term adverse effects on both the mother and the newborn. Additionally, post-caesarean section stress responses are more pronounced, including elevated levels of aldosterone, norepinephrine, and cortisol (Chu and Qiao, 2022). Bodin et al. (2022) also reported that the risk of developing P-PTSD symptoms after a caesarean section is four times higher; Emergency caesarean sections are more likely to lead to P-PTSD than elective caesarean sections (*p* < 0.001) (Carter et al., 2022), with a threefold increase in risk (Dekel et al., 2019). Furthermore, the research reported by Tomsis et al. indicates that the type of caesarean section influences the severity of P-PTSD symptoms through the complete mediating effect of self-control, explaining 38.5% of the variation in symptom levels (Tomsis et al., 2021). During vaginal delivery, mothers perceive weaker dissociation from childbirth compared to caesarean section, have stronger control experiences, and exhibit milder symptom responses to traumatic events.

#### 4.3.3 Core psychological stress covariates

This study found that when high-risk pregnant women exhibit high levels of social support, positive coping, psychological resilience, and low levels of negative coping, P-PTSD symptoms remain at low levels and relatively stable. Some studies suggest that social support is more effective than healthcare in promoting psychological recovery among P-PTSD pregnant women (Chen et al., 2023; Suarez and Yakupova, 2023): this includes family support for actively preparing to welcome a new life, support from experienced other pregnant women or friends, and supportive policies and benefits related to childbirth. Good social support can promote positive coping. The synergistic positive effects of positive coping strategies and high levels of social support following traumatic events were also explored in Zhang Boning et al.’s study (Wang J. et al., 2010). Psychological resilience, as a positive adaptive process when individuals face stressful events, can directly predict P-PTSD (Xia et al., 2014). Previous studies have shown that negative coping can have a positive effect on P-PTSD in high-risk pregnant women, meaning that the more they tend to avoid depression and complain, the higher their P-PTSD levels. Such negative coping may be associated with increased psychological stress, persistent focus on negative thoughts, and passive overthinking of the causes and consequences of events, i.e., rumination (Griffith and Raes, 2015), whose positive predictive effect on PTSD has been reported in previous studies (Xu and Zhu, 2023). Additionally, from a mechanistic perspective, rumination includes two forms: active rumination and intrusive rumination (Calhoun et al., 2000): The former involves purposeful thinking and recollection, which can alleviate psychological stress after trauma; the latter is the passive intrusion of traumatic events, whose cognitive processing mechanisms are entirely different from active rumination. It is a common negative coping mechanism after trauma exposure (Baschnagel et al., 2009), which may reinforce existing negative cognitions, potentially worsen anxiety, tension, and helplessness, and may be associated with PTSD symptoms.

Therefore, it is suggested that screening for P-PTSD at the time of pregnancy risk assessment may help to mitigate perinatal trauma exposure and avoid P-PTSD symptoms; and that focusing on different psychological variables as labour progresses may have a differential impact on the development of P-PTSD. In addition, providing adequate information about perinatal care, training healthcare professionals in trauma-informed care, and avoiding factors that lead to negative evaluations are critical to preventing P-PTSD symptoms.

#### 4.3.4 Cultural and social background

The psychological burden of the “high-risk” label under China’s five-color risk classification system, which may reinforce the “sick role” identity and exacerbate anxiety and loss of control (Ji and Wang, 2026); The central role of family (spouses and both sets of grandparents) in perinatal support, alongside the dual impact of the “zuo yuezi” (confinement) custom, which may simultaneously offer care and generate intergenerational conflict or overprotection (Bai et al., 2025); The psychological pressures associated with the “three-child policy,” the widespread use of assisted reproductive technology leading to multiple pregnancies, and the urban–rural disparities in health insurance coverage and health literacy (Lan et al., 2026).

The advantages of this study are twofold. Firstly, the study focuses on the developmental changes of P-PTSD and its predictors in high-risk pregnant women, which provides new ideas for early warning management of P-PTSD in clinical practice and is of greater significance for clinical guidance; secondly, through prospective longitudinal tracking of the changes of P-PTSD in high-risk pregnant women, the study examines the individual differences in the trend of P-PTSD over time and the differences of psychological stress variables in the different trajectories by examining them comprehensively and completely, from the causes to the results. This will provide high-intensity evidence for the adoption of effective mental health interventions.

There are several limitations of this study: Firstly, the inclusion of the study population has the limitations of cultural background, region, single centre, and short follow-up time may not be conducive to the generalisation of the results. In the future, the diversity of the sample should be increased by expanding the sample size, researching high-risk pregnant women in different regions, and conducting longer follow-up to make the study representative. Second, although the sample size of the smallest trajectory group (*n* = 42) met the minimum recommendations for GBTM, its modest size may limit the precision of parameter estimates in the multinomial logistic regression and the generalizability of findings specific to this subgroup. Future studies with larger samples are needed to validate the three-trajectory solution. Third, this study used the PCL-C, a general civilian scale based on DSM-IV criteria, which was not specifically designed for pregnant women, may lack content validity in capturing specific symptoms during the perinatal period. However, issues regarding its diagnostic validity should be noted. The DSM-5 introduced three major revisions to the PTSD criteria (removal of Criterion A2, addition of the “Negative Cognitions and Emotions” cluster, and separation of the Avoidance and Numbness clusters), which may lead to discrepancies in identification rates between the DSM-IV and DSM-5: on the one hand, the removal of Criterion A2 results in more childbirth events being classified as “traumatic,” which may inflate the prevalence rate under the DSM-5; on the other hand, the PCL-C does not cover the three new symptom items added in DSM-5, which may result in the omission of individuals whose primary complaints involve negative cognitions and emotions. Although the PCL-C has good validity in the perinatal population and a cutoff score of ≥38 offers good sensitivity and specificity, the specific impact of the differences between the two versions of the criteria on the measurement of postpartum PTSD remains unclear. Future studies are recommended to validate population-specific cutoff scores for the PCL-5 in the Chinese perinatal population. Fourth, Self-report bias and the absence of structured clinical interviews. All psychological assessments in this study relied on self-reported scales (PCL-C, CAHS, TCSQ, PSSS, and CD-RISC-10), which are susceptible to recall bias, social desirability effects, and current mood states. Moreover, although the PCL-C with a ≥ 38 cutoff has demonstrated good sensitivity and specificity in pregnant populations, it is a screening tool rather than a diagnostic instrument. The absence of a structured clinical interview (e.g., the Clinician-Administered PTSD Scale, CAPS) means that the positive detection rates reported in this study may not directly equate to clinical diagnoses of PTSD. Future studies should consider incorporating diagnostic interviews to enhance diagnostic precision; Fifth, potential confounding by comorbid depression and anxiety. Perinatal PTSD frequently co-occurs with depression and anxiety, and the symptom overlap between these conditions is well documented. This study did not systematically assess depressive or anxiety symptoms; therefore, we cannot rule out the possibility that some of the P-PTSD symptoms measured in this study may reflect comorbid mood or anxiety disorders rather than PTSD-specific responses. Furthermore, before studying the subjects, we did not exclude or systematically evaluate their previous non-pregnancy-related traumatic events, which may have led to a predisposition to excessive vigilance or accumulated trauma load. Future studies should incorporate a comprehensive lifetime trauma assessment to better control the confounding effects of previous non-pregnancy-related traumas. Additionally, validated depression and anxiety scales should be included to distinguish the unique contributions of each psychological state. Sixth, the cultural specificity of the high-risk classification system. The pregnancy risk classification system used in this study (five-color coding: green, yellow, orange, red, and purple) was developed by the Chinese National Health Commission and is specific to the Chinese health care context. This classification system may differ from high-risk pregnancy definitions used in other countries, which may limit the comparability of our findings with studies conducted in other health care systems. Future international collaborations should consider harmonizing definitions to facilitate cross-country comparisons.

## 5 Conclusion

The present study demonstrated an overall trend of increasing P-PTSD symptoms in high-risk pregnant women from 1 month after diagnosis to 1 month postpartum. The GBTM identified three distinct developmental trajectories: a low-risk stabilization trajectory, a moderate-risk worsening trajectory, and a high-risk fluctuating trajectory. The study identified several key predictive determinants, including socioeconomic stress, neonatal complications, prior trauma exposure, emergency cesarean delivery, and baseline psychological distress. These findings underscore the clinical utility of integrating the P-PTSD trajectory model into a dynamic surveillance framework. By systematically assessing coping resources and stress response characteristics in at-risk populations, clinicians can tailor early intervention programs to interrupt symptom escalation pathways and improve perinatal prognosis.

## Data Availability

The raw data supporting the conclusions of this article will be made available by the authors, without undue reservation.
